# Tricarbonyl­chlorido{*N*-[2-(diphenyl­phosphino)benzyl­idene]benzyl­amine-κ^2^ *N*,*P*}rhenium(I) dichloro­methane solvate

**DOI:** 10.1107/S160053680706802X

**Published:** 2008-01-09

**Authors:** Uwe Monkowius, Manfred Zabel

**Affiliations:** a Johannes Kepler Universität Linz, Institut für Anorganische Chemie, Altenbergerstrasse 69, 4040 Linz, Austria; b Universität Regensburg, Zentrale Analytik, Röntgenstrukturanalyse, Universitätsstrasse 31, 93053 Regensburg, Germany

## Abstract

In the crystal structure of the title compound, [ReCl(C_26_H_22_NP)(CO)_3_]·CH_2_Cl_2_, the Re^I^ atom exhibits a distorted octa­hedral environment defined by a facial arrangement of three carbonyl groups, a Cl atom and an *N*-[2-(diphenyl­phosphino)benzyl­idene]benzyl­amine ligand. The compound crystallizes with one CH_2_Cl_2_ mol­ecule per asymmetric unit. The benzyl­amine ligand and the Re^I^ centre form a non-planar six-membered chelate ring.

## Related literature

For related literature, see: Chen *et al.* (2001 ▶); and Schultz *et al.* (2004 ▶).
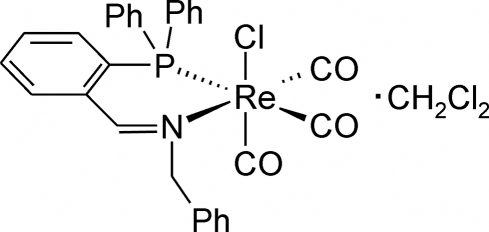


## Experimental

### 

#### Crystal data

[ReCl(C_26_H_22_NP)(CO)_3_]·CH_2_Cl_2_
*M* *_r_* = 770.03
Monoclinic, 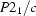

*a* = 16.1971 (12) Å
*b* = 9.1981 (6) Å
*c* = 20.7977 (17) Å
β = 104.820 (9)°
*V* = 2995.4 (4) Å^3^
*Z* = 4
Mo *K*α radiation
μ = 4.41 mm^−1^
*T* = 296 K
0.26 × 0.24 × 0.18 mm

#### Data collection

Stoe IPDS diffractometer
Absorption correction: analytical from crystal shape (**IPDS**; Stoe & Cie, 1998 ▶) *T*_min_ = 0.431, *T*_max_ = 0.612
27893 measured reflections
5789 independent reflections
4647 reflections with *I* > 2σ(*I*)
*R*_int_ = 0.089

#### Refinement

*R*[*F* ^2^ > 2σ(*F* ^2^)] = 0.032
*wR*(*F* ^2^) = 0.081
*S* = 0.99
5789 reflections
355 parameters
H-atom parameters constrained
Δρ_max_ = 1.27 e Å^−3^
Δρ_min_ = −0.56 e Å^−3^

### 

Data collection: *IPDS* (Stoe & Cie, 1998 ▶); cell refinement: *IPDS*; data reduction: *IPDS*; program(s) used to solve structure: *SIR97* (Altomare *et al.*, 1999 ▶); program(s) used to refine structure: *SHELXL97* (Sheldrick, 1997 ▶); molecular graphics: *PLATON* (Spek, 2003 ▶); software used to prepare material for publication: *PLATON*.

## Supplementary Material

Crystal structure: contains datablocks global, I. DOI: 10.1107/S160053680706802X/zl2094sup1.cif

Structure factors: contains datablocks I. DOI: 10.1107/S160053680706802X/zl2094Isup2.hkl

Additional supplementary materials:  crystallographic information; 3D view; checkCIF report

## Figures and Tables

**Table 1:** Hydrogen-bond geometry (Å, °)

*D*—H⋯*A*	*D*—H	H⋯*A*	*D*⋯*A*	*D*—H⋯*A*
C22—H22⋯O3^i^	0.93	2.50	3.327 (8)	149

Symmetry code: (i) 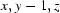.

**Fig. 1.**
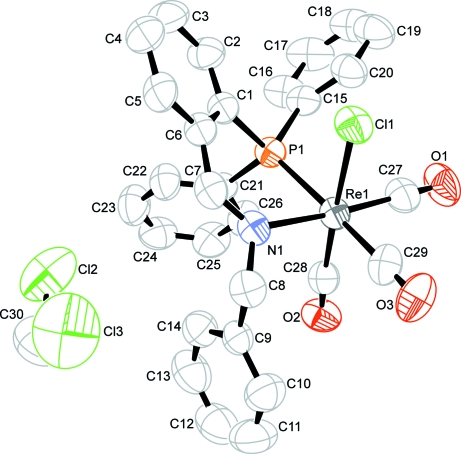
: View of the title compound with the atom numbering scheme. H-atoms have been omitted for clarity. Displacement ellipsoids for non-H atoms are drawn at the 50% probability level.

**Fig. 2.**
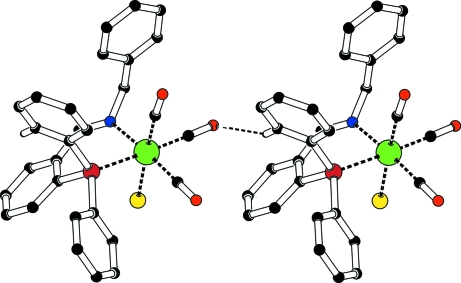
: Excerpt from a cell plot depicting the intermolecular hydrogen-bond between H22 and the carbonyl oxygen atom O3 of a neighboring complex. The H atoms not involved in hydrogen bonding and the dichloromethane molecules have been omitted for clarity.

## supplementary crystallographic information

### Comment

The iminophosphine ligand incorporated in the title compound, 2-(diphenylphosphino)benzylidenebenzylamine, is easily accessible by a condensation reaction of commercially available 2-(diphenylphoshino)benzaldehyde with benzylamine (Schultz *et al.*, 2004). Without further purification the ligand is used for the preparation of the title complex starting from Re(CO)_5_Cl.

The crystals contain one molecule of CH_2_Cl_2_ per unit cell. The coordination geometry at the Re^I^ atom is distorted octahedral with the three carbonyl ligands arranged in a *facial* fashion. Together with the Re^I^ center the *N*,*P*-ligand forms a non-planar, six-membered chelate ring. The N—Re—P bite angle of 83.86 (12)° deviates from the ideal of 90° expected for an octahedron. The Re1—P1 and Re1—N1 bond lengths are 2.4561 (13) and 2.210 (4) Å, respectively. The Re—C bond length *trans* to the P atom (Re1—C29 1.942 (5) Å) is longer than its equivalents *trans* to the chloride and imine nitrogen atoms (Re1—C27 1.926 (6), Re1—C28 1.909 (6) Å) indicating stronger π-backbonding by the coordinated phosphine P atom. In its crystals, the complexes are linked to infinite chains *via* weak C—H···O intermolecular hydrogen bonds with a C···O distance of 3.327 (8) Å and a C22—H22···O3^i^ angle of 149° (symmetry code: (i) *x*, *y* - 1, *z*). In summary, the structural characteristics are very similar to previously reported rhenium complexes of analogous chelating iminophosphine ligands (Chen *et al.*, 2001).

### Experimental

2-(Diphenylphosphino)benzaldehyde (95 mg, 0.33 mmol) and benzylamine (35 mg, 0.33 mmol) were stirred in dichloromethane. After 3 h the solvent was removed under reduced pressure. Degassed toluene (15 ml) and Re(CO)_5_Cl were added and the mixture was refluxed for 2 h under nitrogen atmosphere. After cooling to room temperature, the yellow precipitate was isolated by filtration and dried in vacuum. Recrystallization from dichloromethane/pentane yielded crystals suitable for X-ray crystallography. Yield: 206 mg (0.30 mmol, 91%). ^1^H-NMR (300 MHz, CDCl_3_): δ = 8.33 (s, 1 H, CH=N), 7.63–7.71 (m, 2 H, C_6_H_4_), 7.41–7.60 (m, 10 H, PPh_2_), 7.13–7.27 (m, 5 H, benzyl-Ph), 6.90–6.95 (m, 2 H, C_6_H_4_), 5.29 (s, 2 H, CH_2_); ^31^P{^1^H}-NMR (121 MHz, CDCl_3_): δ = 16.6; MS(EI): *m/z* (%) = 684.8 [*M*]^+^ (17.2), 656.8 [M—CO]^+^ (100), 628.8 [*M*-2CO]^+^ (94.2), 600.9 [*M*-3CO]^+^ (89.4), 564.9 [*M*-3CO-Cl]^+^ (7.3), 509.8 [*M*-3CO-Bn]^+^ (84.1), 91.0 [Bn]^+^ (50.8): EA (C_29_H_22_ClNO_3_PRe) calc.: C 50.84, H 3.24, N 2.04, found: C 50.27, H 3.32, N 1.95.

### Refinement

The data were collected at room temperature. The structure was solved by direct methods (*SIR97*) and refined by full-matrix anisotropic least squares (*SHELXL97*).The H-atoms were placed in geometrically calculated positions and were refined using a riding model with C—H distances of 0.93 or 0.97 Å and isotropic displacement parameters *U*_iso_ equal to 1.2 times *U*_eq_(C).

### Crystal data

**Table:**

[ReCl(C_26_H_22_NP)(CO)_3_]·CH_2_Cl_2_	*F*_000_ = 1504
*M**_r_* = 770.03	Cell parameters were determined by indexing 8000 reflections with I/sigma limit 6.0.
Monoclinic, *P*2_1_/*c*	*D*_x_ = 1.707 Mg m^−^^3^
Hall symbol: -P 2ybc	Mo *K*α radiation λ = 0.71073 Å
*a* = 16.1971 (12) Å	Cell parameters from 8000 reflections
*b* = 9.1981 (6) Å	θ = 2.6–25.9º
*c* = 20.7977 (17) Å	µ = 4.41 mm^−^^1^
β = 104.820 (9)º	*T* = 296 K
*V* = 2995.4 (4) Å^3^	Prism, faint yellow translucent
*Z* = 4	0.26 × 0.24 × 0.18 mm

### Data collection

**Table:**

Stoe IPDS diffractometer	5789 independent reflections
Radiation source: fine-focus sealed tube	4647 reflections with *I* > 2σ(*I*)
Monochromator: graphite	*R*_int_ = 0.090
*T* = 296(1) K	θ_max_ = 25.9º
rotation scans	θ_min_ = 2.6º
Absorption correction: analyticalfrom crystal shape (*IPDS*; Stoe & Cie, 1998)	*h* = −19→19
*T*_min_ = 0.431, *T*_max_ = 0.612	*k* = −11→11
27893 measured reflections	*l* = −25→25

### Refinement

**Table:**

Refinement on *F*^2^	Secondary atom site location: difference Fourier map
Least-squares matrix: full	Hydrogen site location: inferred from neighbouring sites
*R*[*F*^2^ > 2σ(*F*^2^)] = 0.032	H-atom parameters constrained
*wR*(*F*^2^) = 0.081	*w* = 1/[σ^2^(*F*_o_^2^) + (0.0449*P*)^2^] where *P* = (*F*_o_^2^ + 2*F*_c_^2^)/3
*S* = 0.99	(Δ/σ)_max_ = 0.003
5789 reflections	Δρ_max_ = 1.27 e Å^−^^3^
355 parameters	Δρ_min_ = −0.56 e Å^−^^3^
Primary atom site location: structure-invariant direct methods	Extinction correction: none

### Special details

**Table:**

Experimental. Data were collected applying an imaging plate system (Stoe) with the following measurement parameters:Detector distance [mm] 70 Phi movement mode Oscillation Phi incr. [degrees] 1.0 Number of exposures 245 Irradiation / exposure [min] 1.00For a detailed description of the method see: Sheldrick, G.*M*., Paulus, E. Vertesy, *L*. & Hahn, F. (1995) Acta Cryst. B51, 89–98.
Geometry. Bond distances, angles *etc*. have been calculated using the rounded fractional coordinates. All su's are estimated from the variances of the (full) variance-covariance matrix. The cell e.s.d.'s are taken into account in the estimation of distances, angles and torsion angles
Refinement. Refinement of *F*^2^ against ALL reflections. The weighted *R*-factor *wR* and goodness of fit *S* are based on *F*^2^, conventional *R*-factors *R* are based on *F*, with *F* set to zero for negative *F*^2^. The threshold expression of *F*^2^ > σ(*F*^2^) is used only for calculating *R*-factors(gt) *etc*. and is not relevant to the choice of reflections for refinement. *R*-factors based on *F*^2^ are statistically about twice as large as those based on *F*, and *R*- factors based on ALL data will be even larger.

### Fractional atomic coordinates and isotropic or equivalent isotropic displacement parameters (Å^2^)

**Table:**

	*x*	*y*	*z*	*U*_iso_*/*U*_eq_	
Re1	0.76584 (1)	0.11133 (2)	0.96057 (1)	0.0497 (1)	
Cl1	0.87641 (8)	0.10116 (16)	1.06935 (6)	0.0676 (4)	
P1	0.71528 (7)	−0.12810 (14)	0.98633 (6)	0.0497 (4)	
O1	0.6469 (3)	0.2883 (6)	1.0245 (2)	0.0955 (19)	
O2	0.6302 (2)	0.1448 (5)	0.82923 (17)	0.0709 (13)	
O3	0.8385 (3)	0.4027 (5)	0.9260 (3)	0.0873 (18)	
N1	0.8596 (2)	−0.0172 (5)	0.92374 (18)	0.0529 (13)	
C1	0.8066 (3)	−0.2456 (6)	1.0211 (2)	0.0533 (14)	
C2	0.8029 (3)	−0.3498 (7)	1.0682 (3)	0.0700 (19)	
C3	0.8717 (4)	−0.4415 (8)	1.0951 (3)	0.081 (2)	
C4	0.9461 (4)	−0.4281 (7)	1.0746 (3)	0.0762 (19)	
C5	0.9499 (3)	−0.3278 (7)	1.0261 (3)	0.0675 (16)	
C6	0.8821 (3)	−0.2341 (5)	0.9988 (2)	0.0542 (14)	
C7	0.8968 (3)	−0.1364 (6)	0.9471 (2)	0.0574 (18)	
C8	0.8902 (3)	0.0470 (6)	0.8686 (2)	0.0621 (16)	
C9	0.8222 (3)	0.0482 (6)	0.8040 (2)	0.0565 (17)	
C10	0.8126 (5)	0.1643 (8)	0.7620 (3)	0.088 (3)	
C11	0.7522 (7)	0.1629 (12)	0.7011 (4)	0.116 (4)	
C12	0.6989 (6)	0.0464 (12)	0.6828 (4)	0.107 (3)	
C13	0.7084 (5)	−0.0676 (10)	0.7239 (4)	0.098 (3)	
C14	0.7691 (4)	−0.0690 (7)	0.7837 (3)	0.074 (2)	
C15	0.6533 (3)	−0.1358 (6)	1.0491 (2)	0.0591 (18)	
C16	0.5835 (3)	−0.2249 (8)	1.0432 (3)	0.079 (2)	
C17	0.5405 (4)	−0.2269 (9)	1.0929 (4)	0.093 (3)	
C18	0.5671 (4)	−0.1411 (10)	1.1476 (4)	0.098 (3)	
C19	0.6368 (5)	−0.0521 (9)	1.1547 (3)	0.092 (3)	
C20	0.6788 (4)	−0.0498 (7)	1.1043 (3)	0.0739 (19)	
C21	0.6505 (3)	−0.2346 (5)	0.9178 (2)	0.0526 (14)	
C22	0.6740 (4)	−0.3688 (6)	0.8994 (3)	0.0706 (19)	
C23	0.6192 (4)	−0.4487 (8)	0.8502 (3)	0.089 (2)	
C24	0.5409 (4)	−0.3950 (7)	0.8180 (3)	0.082 (2)	
C25	0.5170 (4)	−0.2618 (8)	0.8343 (3)	0.080 (2)	
C26	0.5704 (3)	−0.1808 (7)	0.8830 (3)	0.0698 (17)	
C27	0.6910 (3)	0.2220 (7)	1.0005 (3)	0.0671 (19)	
C28	0.6818 (3)	0.1268 (5)	0.8777 (3)	0.0570 (16)	
C29	0.8126 (3)	0.2947 (6)	0.9399 (3)	0.0640 (16)	
Cl2	0.8504 (2)	−0.4410 (3)	0.78245 (18)	0.1549 (13)	
Cl3	0.9854 (2)	−0.2588 (5)	0.7679 (2)	0.197 (2)	
C30	0.8967 (8)	−0.3533 (13)	0.7263 (5)	0.149 (5)	
H2	0.75310	−0.35900	1.08240	0.0840*	
H3	0.86760	−0.51100	1.12660	0.0970*	
H4	0.99320	−0.48610	1.09330	0.0910*	
H5	0.99910	−0.32240	1.01110	0.0810*	
H7	0.94020	−0.16560	0.92810	0.0690*	
H8A	0.93880	−0.00830	0.86290	0.0740*	
H8B	0.90910	0.14580	0.88020	0.0740*	
H10	0.84700	0.24580	0.77440	0.1060*	
H11	0.74790	0.24190	0.67250	0.1390*	
H12	0.65700	0.04650	0.64270	0.1280*	
H13	0.67310	−0.14810	0.71160	0.1180*	
H14	0.77430	−0.15040	0.81090	0.0880*	
H16	0.56520	−0.28370	1.00590	0.0940*	
H17	0.49330	−0.28700	1.08890	0.1110*	
H18	0.53770	−0.14280	1.18060	0.1170*	
H19	0.65550	0.00530	1.19240	0.1110*	
H20	0.72540	0.01160	1.10820	0.0890*	
H22	0.72740	−0.40640	0.92040	0.0850*	
H23	0.63590	−0.53990	0.83880	0.1070*	
H24	0.50420	−0.44970	0.78520	0.0980*	
H25	0.46380	−0.22480	0.81220	0.0960*	
H26	0.55330	−0.08890	0.89310	0.0840*	
H30A	0.91260	−0.42430	0.69720	0.1790*	
H30B	0.85580	−0.28690	0.69910	0.1790*	

### Atomic displacement parameters (Å^2^)

**Table:**

	*U*^11^	*U*^22^	*U*^33^	*U*^12^	*U*^13^	*U*^23^
Re1	0.0361 (1)	0.0583 (1)	0.0510 (1)	0.0058 (1)	0.0046 (1)	−0.0049 (1)
Cl1	0.0481 (6)	0.0854 (9)	0.0596 (6)	0.0045 (6)	−0.0038 (5)	−0.0101 (6)
P1	0.0351 (5)	0.0620 (8)	0.0503 (6)	0.0073 (5)	0.0076 (4)	0.0006 (5)
O1	0.086 (3)	0.117 (4)	0.083 (3)	0.042 (3)	0.021 (2)	−0.023 (2)
O2	0.0459 (17)	0.103 (3)	0.0553 (19)	0.0123 (18)	−0.0024 (15)	0.0056 (18)
O3	0.064 (2)	0.063 (3)	0.131 (4)	0.004 (2)	0.018 (2)	0.006 (2)
N1	0.0376 (18)	0.065 (3)	0.054 (2)	0.0041 (18)	0.0078 (15)	−0.0065 (17)
C1	0.040 (2)	0.064 (3)	0.051 (2)	0.010 (2)	0.0028 (17)	−0.0034 (19)
C2	0.059 (3)	0.084 (4)	0.067 (3)	0.016 (3)	0.016 (2)	0.014 (3)
C3	0.076 (4)	0.091 (4)	0.071 (3)	0.019 (3)	0.011 (3)	0.022 (3)
C4	0.060 (3)	0.084 (4)	0.075 (3)	0.025 (3)	0.000 (3)	0.007 (3)
C5	0.045 (2)	0.078 (3)	0.074 (3)	0.020 (3)	0.005 (2)	−0.007 (3)
C6	0.040 (2)	0.064 (3)	0.054 (2)	0.008 (2)	0.0034 (18)	−0.004 (2)
C7	0.036 (2)	0.072 (4)	0.064 (3)	0.006 (2)	0.0126 (19)	−0.010 (2)
C8	0.048 (2)	0.070 (3)	0.072 (3)	−0.006 (2)	0.022 (2)	−0.003 (2)
C9	0.054 (3)	0.060 (3)	0.061 (3)	0.007 (2)	0.025 (2)	−0.003 (2)
C10	0.099 (5)	0.078 (4)	0.095 (4)	0.004 (4)	0.037 (4)	0.014 (4)
C11	0.140 (7)	0.127 (7)	0.085 (5)	0.047 (6)	0.037 (5)	0.042 (5)
C12	0.101 (5)	0.145 (8)	0.069 (4)	0.039 (6)	0.011 (4)	−0.002 (5)
C13	0.098 (5)	0.111 (6)	0.079 (4)	−0.003 (4)	0.010 (4)	−0.028 (4)
C14	0.079 (4)	0.071 (4)	0.066 (3)	−0.001 (3)	0.010 (3)	−0.007 (3)
C15	0.041 (2)	0.079 (4)	0.058 (3)	0.013 (2)	0.0141 (19)	0.010 (2)
C16	0.050 (3)	0.115 (5)	0.071 (3)	−0.004 (3)	0.016 (2)	0.013 (3)
C17	0.049 (3)	0.146 (7)	0.085 (4)	0.003 (3)	0.020 (3)	0.029 (4)
C18	0.066 (4)	0.158 (7)	0.078 (4)	0.041 (4)	0.035 (3)	0.031 (4)
C19	0.087 (5)	0.129 (6)	0.069 (4)	0.032 (4)	0.034 (3)	0.006 (4)
C20	0.068 (3)	0.093 (4)	0.064 (3)	0.015 (3)	0.023 (3)	0.000 (3)
C21	0.044 (2)	0.058 (3)	0.053 (2)	0.002 (2)	0.0072 (18)	0.0029 (19)
C22	0.060 (3)	0.070 (4)	0.074 (3)	0.012 (3)	0.003 (2)	−0.009 (2)
C23	0.091 (4)	0.072 (4)	0.094 (4)	0.004 (4)	0.006 (4)	−0.020 (3)
C24	0.080 (4)	0.086 (4)	0.067 (3)	−0.018 (3)	−0.002 (3)	0.001 (3)
C25	0.052 (3)	0.093 (5)	0.080 (4)	0.001 (3)	−0.008 (3)	0.002 (3)
C26	0.049 (3)	0.072 (3)	0.079 (3)	0.009 (3)	−0.001 (2)	−0.003 (3)
C27	0.055 (3)	0.080 (4)	0.059 (3)	0.010 (3)	0.001 (2)	−0.006 (2)
C28	0.048 (2)	0.060 (3)	0.065 (3)	0.004 (2)	0.018 (2)	−0.002 (2)
C29	0.041 (2)	0.062 (3)	0.083 (3)	0.008 (2)	0.005 (2)	−0.005 (3)
Cl2	0.146 (2)	0.1108 (18)	0.195 (3)	−0.0005 (17)	0.020 (2)	0.0389 (19)
Cl3	0.110 (2)	0.214 (4)	0.268 (5)	−0.026 (2)	0.053 (2)	−0.016 (3)
C30	0.168 (10)	0.164 (10)	0.104 (6)	−0.014 (8)	0.013 (6)	−0.014 (6)

### Geometric parameters (Å, °)

**Table:**

Re1—Cl1	2.5024 (13)	C17—C18	1.361 (12)
Re1—P1	2.4561 (13)	C18—C19	1.372 (11)
Re1—N1	2.210 (4)	C19—C20	1.388 (10)
Re1—C27	1.926 (6)	C21—C26	1.404 (7)
Re1—C28	1.909 (6)	C21—C22	1.375 (7)
Re1—C29	1.942 (5)	C22—C23	1.382 (9)
Cl2—C30	1.739 (12)	C23—C24	1.366 (9)
Cl3—C30	1.712 (13)	C24—C25	1.354 (10)
P1—C21	1.824 (5)	C25—C26	1.372 (9)
P1—C1	1.825 (5)	C2—H2	0.9300
P1—C15	1.841 (5)	C3—H3	0.9300
O1—C27	1.146 (8)	C4—H4	0.9300
O2—C28	1.145 (7)	C5—H5	0.9300
O3—C29	1.144 (7)	C7—H7	0.9300
N1—C7	1.285 (7)	C8—H8B	0.9700
N1—C8	1.483 (6)	C8—H8A	0.9700
C1—C6	1.418 (7)	C10—H10	0.9300
C1—C2	1.383 (8)	C11—H11	0.9300
C2—C3	1.396 (9)	C12—H12	0.9300
C3—C4	1.383 (9)	C13—H13	0.9300
C4—C5	1.380 (9)	C14—H14	0.9300
C5—C6	1.397 (7)	C16—H16	0.9300
C6—C7	1.467 (6)	C17—H17	0.9300
C8—C9	1.504 (6)	C18—H18	0.9300
C9—C14	1.376 (8)	C19—H19	0.9300
C9—C10	1.363 (9)	C20—H20	0.9300
C10—C11	1.388 (11)	C22—H22	0.9300
C11—C12	1.368 (15)	C23—H23	0.9300
C12—C13	1.337 (13)	C24—H24	0.9300
C13—C14	1.374 (10)	C25—H25	0.9300
C15—C16	1.376 (8)	C26—H26	0.9300
C15—C20	1.368 (8)	C30—H30A	0.9700
C16—C17	1.386 (9)	C30—H30B	0.9700
			
Re1···C14	4.048 (6)	C29···C8	3.150 (8)
Re1···H20	3.4200	C29···Cl1	3.172 (6)
Cl1···P1	3.4546 (19)	C29···C28	2.673 (8)
Cl1···N1	3.164 (4)	C29···C27	2.680 (8)
Cl1···C1	3.447 (6)	C29···N1	3.009 (7)
Cl1···C6	3.426 (5)	C1···H22	2.6200
Cl1···C7	3.432 (5)	C2···H30B^iv^	2.9200
Cl1···C27	3.179 (6)	C2···H22	3.0500
Cl1···C29	3.172 (6)	C3···H30B^iv^	3.0700
Cl2···O3^i^	3.363 (7)	C5···H8B^iii^	3.0900
Cl3···C10^ii^	3.554 (9)	C6···H22	3.0600
Cl1···H20	2.8900	C7···H14	3.0200
Cl1···H8A^iii^	3.0800	C11···H3^x^	3.0500
Cl1···H30A^iv^	3.0400	C12···H2^x^	3.0100
Cl1···H7^iii^	3.0200	C15···H2	2.6000
Cl2···H10^i^	2.8900	C16···H2	2.9300
Cl2···H14	3.0600	C17···H13^iv^	3.0500
Cl3···H10^ii^	3.0600	C18···H13^iv^	2.7000
P1···C7	3.247 (5)	C18···H26^v^	2.8500
P1···C27	3.267 (7)	C19···H13^iv^	3.0000
P1···Cl1	3.4546 (19)	C21···H16	2.6000
P1···N1	3.123 (4)	C24···H18^x^	2.8700
P1···C28	3.205 (5)	C26···H16	2.7500
O2···C13	3.412 (9)	C27···H20	2.9000
O2···C9	3.402 (6)	C28···H26	2.9500
O2···C14	3.304 (8)	C29···H22^vi^	3.0600
O2···C18^v^	3.352 (8)	C29···H8B	2.6200
O3···C22^vi^	3.327 (8)	C29···H5^iii^	2.9700
O3···Cl2^vi^	3.363 (7)	H2···C15	2.6000
O3···C5^iii^	3.386 (7)	H2···C16	2.9300
O1···H17^v^	2.8200	H2···C12^iv^	3.0100
O1···H12^vii^	2.8600	H3···C11^iv^	3.0500
O2···H18^v^	2.6700	H5···O3^iii^	2.7200
O2···H23^vi^	2.9100	H5···C29^iii^	2.9700
O2···H24^viii^	2.9200	H5···H7	2.2600
O3···H8B	2.8900	H7···H8A	1.9800
O3···H22^vi^	2.5000	H7···Cl1^iii^	3.0200
O3···H5^iii^	2.7200	H7···H5	2.2600
N1···P1	3.123 (4)	H8A···Cl1^iii^	3.0800
N1···Cl1	3.164 (4)	H8A···H7	1.9800
N1···C1	3.184 (6)	H8B···C29	2.6200
N1···C28	3.091 (6)	H8B···O3	2.8900
N1···C29	3.009 (7)	H8B···H10	2.3600
N1···H14	2.6900	H8B···C5^iii^	3.0900
C5···O3^iii^	3.386 (7)	H10···Cl3^ix^	3.0600
C6···Cl1	3.426 (5)	H10···Cl2^vi^	2.8900
C7···C14	3.552 (7)	H10···H8B	2.3600
C9···O2	3.402 (6)	H12···O1^xi^	2.8600
C9···C28	3.135 (7)	H13···C18^x^	2.7000
C10···Cl3^ix^	3.554 (9)	H13···C19^x^	3.0000
C13···O2	3.412 (9)	H13···C17^x^	3.0500
C14···Re1	4.048 (6)	H14···N1	2.6900
C14···C28	3.237 (8)	H14···C7	3.0200
C14···C7	3.552 (7)	H14···Cl2	3.0600
C14···O2	3.304 (8)	H16···C26	2.7500
C16···C26	3.309 (9)	H16···C21	2.6000
C18···O2^v^	3.352 (8)	H17···O1^v^	2.8200
C20···C27	3.342 (9)	H18···O2^v^	2.6700
C22···O3^i^	3.327 (8)	H18···C24^iv^	2.8700
C26···C28	3.373 (8)	H18···H24^iv^	2.5200
C26···C16	3.309 (9)	H20···Re1	3.4200
C27···P1	3.267 (7)	H20···C27	2.9000
C27···C15	3.541 (8)	H20···Cl1	2.8900
C27···C28	2.668 (8)	H22···C1	2.6200
C27···C20	3.342 (9)	H22···C2	3.0500
C27···Cl1	3.179 (6)	H22···O3^i^	2.5000
C27···C29	2.680 (8)	H22···C29^i^	3.0600
C28···C8	3.507 (7)	H22···C6	3.0600
C28···C14	3.237 (8)	H23···O2^i^	2.9100
C28···P1	3.205 (5)	H24···H18^x^	2.5200
C28···C26	3.373 (8)	H24···O2^xii^	2.9200
C28···C21	3.496 (7)	H26···C18^v^	2.8500
C28···C9	3.135 (7)	H26···C28	2.9500
C28···C27	2.668 (8)	H30A···Cl1^x^	3.0400
C28···C29	2.673 (8)	H30B···C2^x^	2.9200
C28···N1	3.091 (6)	H30B···C3^x^	3.0700
			
Cl1—Re1—P1	88.32 (4)	C23—C24—C25	119.6 (6)
Cl1—Re1—N1	84.09 (10)	C24—C25—C26	120.7 (6)
Cl1—Re1—C27	90.79 (18)	C21—C26—C25	121.0 (6)
Cl1—Re1—C28	177.87 (14)	Re1—C27—O1	179.6 (4)
Cl1—Re1—C29	90.18 (18)	Re1—C28—O2	175.6 (4)
P1—Re1—N1	83.86 (12)	Re1—C29—O3	178.1 (6)
P1—Re1—C27	95.63 (19)	C1—C2—H2	119.00
P1—Re1—C28	93.63 (15)	C3—C2—H2	119.00
P1—Re1—C29	176.34 (16)	C2—C3—H3	120.00
N1—Re1—C27	174.9 (2)	C4—C3—H3	120.00
N1—Re1—C28	96.98 (18)	C3—C4—H4	120.00
N1—Re1—C29	92.66 (19)	C5—C4—H4	120.00
C27—Re1—C28	88.2 (2)	C4—C5—H5	119.00
C27—Re1—C29	87.7 (2)	C6—C5—H5	119.00
C28—Re1—C29	87.9 (2)	N1—C7—H7	115.00
Re1—P1—C1	109.62 (17)	C6—C7—H7	115.00
Re1—P1—C15	117.52 (18)	N1—C8—H8A	109.00
Re1—P1—C21	117.93 (15)	N1—C8—H8B	109.00
C1—P1—C15	103.3 (2)	C9—C8—H8A	109.00
C1—P1—C21	103.7 (2)	C9—C8—H8B	109.00
C15—P1—C21	103.0 (2)	H8A—C8—H8B	108.00
Re1—N1—C7	128.5 (3)	C9—C10—H10	119.00
Re1—N1—C8	116.7 (3)	C11—C10—H10	120.00
C7—N1—C8	114.5 (4)	C10—C11—H11	120.00
P1—C1—C2	120.9 (4)	C12—C11—H11	120.00
P1—C1—C6	120.4 (4)	C11—C12—H12	121.00
C2—C1—C6	118.7 (5)	C13—C12—H12	121.00
C1—C2—C3	121.9 (5)	C12—C13—H13	119.00
C2—C3—C4	119.5 (6)	C14—C13—H13	119.00
C3—C4—C5	119.4 (6)	C9—C14—H14	119.00
C4—C5—C6	122.2 (5)	C13—C14—H14	119.00
C1—C6—C5	118.4 (4)	C15—C16—H16	120.00
C1—C6—C7	126.9 (4)	C17—C16—H16	120.00
C5—C6—C7	114.7 (4)	C16—C17—H17	120.00
N1—C7—C6	130.7 (4)	C18—C17—H17	120.00
N1—C8—C9	112.4 (4)	C17—C18—H18	120.00
C8—C9—C10	121.0 (5)	C19—C18—H18	120.00
C8—C9—C14	121.7 (5)	C18—C19—H19	121.00
C10—C9—C14	117.2 (5)	C20—C19—H19	121.00
C9—C10—C11	121.0 (7)	C15—C20—H20	119.00
C10—C11—C12	120.6 (9)	C19—C20—H20	119.00
C11—C12—C13	118.4 (8)	C21—C22—H22	120.00
C12—C13—C14	121.7 (8)	C23—C22—H22	120.00
C9—C14—C13	121.1 (6)	C22—C23—H23	120.00
P1—C15—C16	123.0 (4)	C24—C23—H23	120.00
P1—C15—C20	118.1 (4)	C23—C24—H24	120.00
C16—C15—C20	118.9 (5)	C25—C24—H24	120.00
C15—C16—C17	120.0 (6)	C24—C25—H25	120.00
C16—C17—C18	120.2 (7)	C26—C25—H25	120.00
C17—C18—C19	120.8 (7)	C21—C26—H26	120.00
C18—C19—C20	118.5 (7)	C25—C26—H26	120.00
C15—C20—C19	121.6 (6)	Cl2—C30—Cl3	110.3 (6)
P1—C21—C22	123.7 (4)	Cl2—C30—H30A	110.00
P1—C21—C26	119.0 (4)	Cl2—C30—H30B	110.00
C22—C21—C26	117.3 (5)	Cl3—C30—H30A	110.00
C21—C22—C23	120.8 (6)	Cl3—C30—H30B	110.00
C22—C23—C24	120.7 (6)	H30A—C30—H30B	108.00
			
Cl1—Re1—P1—C1	−42.83 (15)	Re1—N1—C7—C6	8.8 (8)
N1—Re1—P1—C1	41.41 (18)	C2—C1—C6—C7	176.7 (5)
C27—Re1—P1—C1	−133.5 (2)	C2—C1—C6—C5	−0.7 (7)
C28—Re1—P1—C1	138.1 (2)	P1—C1—C2—C3	179.5 (5)
Cl1—Re1—P1—C15	74.62 (17)	C6—C1—C2—C3	1.2 (8)
N1—Re1—P1—C15	158.86 (19)	P1—C1—C6—C5	−179.0 (4)
C27—Re1—P1—C15	−16.0 (2)	P1—C1—C6—C7	−1.6 (7)
C28—Re1—P1—C15	−104.5 (2)	C1—C2—C3—C4	0.3 (10)
Cl1—Re1—P1—C21	−161.14 (19)	C2—C3—C4—C5	−2.4 (10)
N1—Re1—P1—C21	−76.9 (2)	C3—C4—C5—C6	2.9 (9)
C27—Re1—P1—C21	108.2 (3)	C4—C5—C6—C7	−179.1 (5)
C28—Re1—P1—C21	19.7 (2)	C4—C5—C6—C1	−1.3 (8)
Cl1—Re1—N1—C7	53.1 (4)	C5—C6—C7—N1	−161.0 (5)
P1—Re1—N1—C7	−35.9 (4)	C1—C6—C7—N1	21.6 (8)
C28—Re1—N1—C7	−128.8 (4)	N1—C8—C9—C10	139.8 (6)
C29—Re1—N1—C7	143.0 (4)	N1—C8—C9—C14	−42.4 (7)
Cl1—Re1—N1—C8	−121.0 (3)	C14—C9—C10—C11	−0.6 (10)
P1—Re1—N1—C8	150.1 (3)	C8—C9—C10—C11	177.3 (7)
C28—Re1—N1—C8	57.2 (3)	C10—C9—C14—C13	−0.8 (9)
C29—Re1—N1—C8	−31.0 (3)	C8—C9—C14—C13	−178.6 (6)
Re1—P1—C1—C2	147.4 (4)	C9—C10—C11—C12	2.2 (14)
C15—P1—C1—C2	21.4 (5)	C10—C11—C12—C13	−2.2 (15)
C21—P1—C1—C2	−85.8 (5)	C11—C12—C13—C14	0.9 (14)
Re1—P1—C1—C6	−34.3 (4)	C12—C13—C14—C9	0.7 (12)
C15—P1—C1—C6	−160.4 (4)	P1—C15—C16—C17	179.0 (5)
C21—P1—C1—C6	92.5 (4)	C16—C15—C20—C19	1.0 (9)
C1—P1—C15—C20	80.7 (5)	C20—C15—C16—C17	−0.3 (9)
C21—P1—C15—C20	−171.5 (4)	P1—C15—C20—C19	−178.3 (5)
Re1—P1—C15—C20	−40.1 (5)	C15—C16—C17—C18	−0.1 (11)
Re1—P1—C21—C22	118.7 (4)	C16—C17—C18—C19	−0.3 (12)
C1—P1—C21—C22	−2.7 (5)	C17—C18—C19—C20	1.0 (12)
C15—P1—C21—C22	−110.1 (5)	C18—C19—C20—C15	−1.4 (11)
Re1—P1—C21—C26	−63.9 (4)	P1—C21—C22—C23	175.2 (5)
C1—P1—C21—C26	174.7 (4)	C26—C21—C22—C23	−2.2 (8)
C15—P1—C21—C26	67.3 (4)	P1—C21—C26—C25	−175.3 (5)
C21—P1—C15—C16	9.2 (5)	C22—C21—C26—C25	2.2 (8)
Re1—P1—C15—C16	140.6 (4)	C21—C22—C23—C24	1.0 (10)
C1—P1—C15—C16	−98.6 (5)	C22—C23—C24—C25	0.3 (10)
C7—N1—C8—C9	114.7 (5)	C23—C24—C25—C26	−0.4 (10)
Re1—N1—C8—C9	−70.4 (5)	C24—C25—C26—C21	−0.9 (9)
C8—N1—C7—C6	−177.1 (5)		

Symmetry codes: (i) *x*, *y*−1, *z*; (ii) −*x*+2, *y*−1/2, −*z*+3/2; (iii) −*x*+2, −*y*, −*z*+2; (iv) *x*, −*y*−1/2, *z*+1/2; (v) −*x*+1, −*y*, −*z*+2; (vi) *x*, *y*+1, *z*; (vii) *x*, −*y*+1/2, *z*+1/2; (viii) −*x*+1, *y*+1/2, −*z*+3/2; (ix) −*x*+2, *y*+1/2, −*z*+3/2; (x) *x*, −*y*−1/2, *z*−1/2; (xi) *x*, −*y*+1/2, *z*−1/2; (xii) −*x*+1, *y*−1/2, −*z*+3/2.

### Hydrogen-bond geometry (Å, °)

**Table:**

*D*—H···*A*	*D*—H	H···*A*	*D*···*A*	*D*—H···*A*
C22—H22···O3^i^	0.93	2.50	3.327 (8)	149

Symmetry codes: (i) *x*, *y*−1, *z*.
